# Depression, Adherence, and Functionality in Patients Undergoing Hemodialysis

**DOI:** 10.7759/cureus.21872

**Published:** 2022-02-03

**Authors:** Zoi-Maria Fotaraki, Georgia Gerogianni, Georgios Vasilopoulos, Maria Polikandrioti, Natalia Giannakopoulou, Victoria Alikari

**Affiliations:** 1 Department of Nursing, Postgraduate Program "Applied Clinical Nursing", University of West Attica, Athens, GRC; 2 Internal Medicine, Attikon General Hospital, Athens, GRC

**Keywords:** gr-smaq-hd, zung, hemodialysis, functionality, depression, adherence

## Abstract

Background

Patients undergoing hemodialysis face multiple problems such as difficulties in performing daily activities, low functional capacity, non-adherence to the hemodialysis regimen, and depressive symptoms that lead to poor health outcomes. The present study aimed to assess the levels of depression, adherence, and functionality in patients undergoing hemodialysis, as well as the association between the above variables.

Materials and methods

In this cross-sectional study, 100 patients undergoing hemodialysis from a private hospital in Athens participated. Data were collected via the Zung Self-Rating Depression Scale, the Barthel Scale/Index, and the Greek Simplified Medication Adherence Questionnaire-Hemodialysis for the evaluation of patients’ depression, functionality, and adherence to hemodialysis regimen, respectively. In addition, sociodemographic and clinical characteristics were recorded. The study was conducted during the period of December 2020 to February 2021. IBM SPSS Statistics for Windows, Version 25.0 (Released 2017. IBM Corp., Armonk, New York) was used for the statistical analysis of the data. The statistical significance level was set up at 0.05.

Results

Of the patients, 50% scored < 38 (possible range 20-80) in the scale pof depression, and 25% of patients scored < 34. Regarding adherence, the median value was 7 (IQR: 7-7) while 77% scored 7 (possible range of 0-8). Regarding functionality, mild dependence (score 91-99) was referred by 77% of the participants, moderate dependence (score 61-90) by 17%, and severe dependence (score 21-60) was referred by 6% of the patients. A statistically significant negative association emerged between depression and functionality (r= -0.342, p=0.001) while no significant association arised between depression and adherence (r= 0.021,p=0.836) as well as between adherence and functionality (r = 0.078, p = 0.439). Statistical significantly higher scores of depression were seen in women (β = 3.65, p = 0.001) and elderly >70 years old (β = 3.51, CI=0.09-6.93, p = 0.044). Statistically significantly lower functionality were referred by patients >70 years old (β = -13.58, CI: -21.68-5.49, p = 0.001) and by patients with high depression score (β= -0.62, 95% CI: -1.06-0.20, p = 0.005).

Conclusion

Patients experienced moderate to low levels of depression and high levels of adherence and functionality. The higher the functionality scores, the lower the depression scores. Demographic characteristics, such as age and gender, seem to be significant predictors of depression and functionality.

## Introduction

In recent decades, the incidence of chronic kidney disease has increased significantly, resulting in an increase in the number of people undergoing hemodialysis (HD). From 2001 to 2020, the prevalence of chronic kidney disease raised steadily in all countries. In 2018, the number of end-stage renal disease cases in the United States was 785.883, while in 2017 it was 761.227 (increase rate of 3.2%). The largest incidence of end-stage renal disease (> 400 per million people) was presented in Mexico (Jalisco region), Taiwan, and Hungary [1]. In Greece, about 11,000 patients undergo HD and about 800 undergo peritoneal dialysis [2].

Patients undergoing HD experience a variety of difficulties arising from the biological dimension of the disease, such as the need to follow a specific diet, the restriction of fluid intake, physical and cognitive impairment, problems related to the arteriovenous fistula, and bone metabolism. In addition, due to physical and cognitive impairment, patients undergoing HD experience a loss of function in daily life and a decrease in the degree of independence [3]. Patients experience a wide range of clinical disorders such as fatigue, physical decline, pain, discomfort, limitations in physical activity, weakness, lack of self-care, feelings of insufficiency, and negative dispositions. The progressive metabolic changes that accompany chronic kidney disease negatively affect patients' energy and activity levels, sleep, and eating behavior, and general health status. It is not surprising that the increased morbidity and mortality of HD patients is in part due to the declined functional status. Uraemic neuropathy, hypoalbuminemia, and hypotensive episodes during HD sessions contribute to reduced functionality and affect the ability to perform activities of daily living and self-management such as dressing, feeding, toileting, or bathing [4]. Levels of functionality and independence are significantly reduced, especially in the elderly after HD initiation. It should also be noted that patients undergoing HD are on average over 80 years old, which impairs functional capacity. The condition worsens if the comorbidities that often accompany uremia, such as uremic encephalopathy or electrolytic disorders, and hypertension, are taken into account. According to Burns et al., age is inversely correlated to physical function and vitality [5].

Lack of energy due to reduced physical fitness can cause depression and mood disorders. Patients undergoing HD present high rates of depressive symptoms. Numerous factors cause depressive behavior in patients undergoing HD. As is well known, patients undergoing HD have to visit the hospital or the HD unit usually three times a week for about four hours [6]. Patients experience intense frustration and anxiety about the future and imminent death. The above in combination with changes in every domain of human condition (personal, family, professional), the changes in self-esteem, and reduction of personal time results in decreased levels of self-esteem, withdrawal, and social isolation. As a result, HD patients conclude that their lives revolve around the home and the hospital and they do not have the opportunity to spend time on social relationships, family, or activities that used to please them [7]. The depression symptoms they experience are often undiagnosed either because HD patients themselves are unwilling to ask for help or the symptoms of depression are hidden from health professionals because professionals focus more on the patient's physical discomfort [8].

For the successful treatment of these health issues, patients’ adherence to the HD regimen is essential. According to the WHO, the definition of adherence concerns the extent to which the individual behaves correctly, in accordance with the instructions of the health professional regarding the medications, the proposed diet, or the change of lifestyle [9]. For the HD patients, adherence includes not only the above dimensions but also the mandatory attendance at the HD session for three days /week, and restriction of fluid intake. The National Kidney Foundation refers that 50% of patients undergoing HD do not adhere to their HD regimen [10] while the rates of non-adherence in the medication range between 12.5-98.6% leading to a greater proportion of morbidity and mortality [11]. The reasons for non-adherence to the HD regimen depend directly on the disease as well as on the complexity of the HD regimen and the increased burden of co-morbidity [12]. Socio-demographic factors such as age, race, low income, cultural factors, or low health literacy contribute to non-adherence, especially in poor and ethnic minority populations. It is understood that the successful effort to improve the level of HD patient adherence depends on factors related to HD patients, the HD regimen, the inveteracy of the kidney disease, socioeconomic criteria, and patients' perceptions of treatment [13]. Αt this point it should be emphasized that adherence levels can be studied through objective measurements (levels of serum phosphorus or potassium and body weight before the HD session). However, there is no perfectly appropriate way to measure all dimensions of adherence in HD.

It seems that the above variables (functionality, depression, adherence,) significantly affect patients undergoing HD. Thus, the aim of the study was to investigate the levels of functionality, depression, and adherence in patients undergoing HD as well as the correlation between the three variables. This is the first study exploring the aforementioned variables among HD patients not only in Greece but internationally.

## Materials and methods

Study design

This is a cross-sectional study with a convenience sample. The study included patients undergoing HD in an HD unit of Bioclinic Hospital, Athens, during the period December 2020-February 2021. All (Ν= 121) HD patients were called to participate in the study. Of these, 110 accepted and, finally, 100 were found eligible (response rate 82,6%). Inclusion criteria were: (1) undergoing HD for at least 1 year, and (2) age above 18 years old. Patients with eye disorders, illiterate patients, and those with diagnosed psychiatric diseases were excluded (as the scales are self-reported questionnaires and are not used for the diagnosis of depression). The questionnaires were distributed by the researchers during the interval of the HD session.

The instruments

The Zung Self-Rating Depression Scale (SGS) was used to assess depression in patients undergoing HD [14]. The scale consists of 20 items that evaluate how patients felt during the past seven days. Participants answered each item on a four-point Likert-type scale (a little of the time - all of the time). In five questions the score is reversed. The final score comes from the sum of the scores on all questions and ranges from 20-80 (< 50 Normal, 50-59 Mild Depression, 60-69 Moderate Depression, 70 Severe Depression). The higher the scores, the higher the levels of depression [15]. The scale has been widely used in several studies [16]. In this study, the Greek version was used [17].

The Barthel index (BI) was used to assess the functionality of patients undergoing HD [18]. The scale is a simple way of measuring the patients’ ability to function and their degree of independence. It consists of 10 variables that assess the patients’ daily activities (feeding, bathing, personal care, dressing, defecation, urination, use of the toilet, transfer from the wheelchair to the bed, and vice versa, movement on flat surfaces, and movement on stairs). The patient's overall performance is the sum of his scores in the 10 activities assessed. Scores 0-20 indicate Total Dependence, 21-60 Severe Dependence, 61-90 Moderate Dependence, and 91-99 indicate Mild Dependence. The scale has been used in studies [19,20] while the Greek version [21] has great internal consistency (0.93-0.95).

The Greek Simplified Medication Adherence Questionnaire for hemodialysis patients (GR-SMAQ-HD) was used to assess the adherence of patients undergoing HD [22]. The scale was constructed by Alikari et al. in 2017 [22]. It includes eight questions, grouped into three factors: Medication Adherence (score 0-4), Attendance at HD session (score 0-2), and Diet /Fluid Restriction (score 0-2). The total score ranges from 0-8. Higher scores indicate greater adherence. The scale has been used in several studies in Greece [13,23].

Also, socio-demographic characteristics (age, gender, marital status, educational level, job) and data regarding the underlying disease and the current health status of the participants were collected.

Statistical analysis

The normality of the data was tested with the Kolmogorov-Smirnov test. In cases the data didn’t follow the normal distribution, continuous data were presented with median and interquartile range (IQR). Categorical data were presented with absolute and relative (%) frequencies. The association between scales (depression, functionality, adherence) was assessed with Spearman’s rho correlation coefficient. In addition, multiple linear regression was performed to assess the impact of patients’ characteristics, functionality, and adherence (independent factors) on their depression (dependent variable) as well as the impact of patients’ characteristics and depression (independent variables) on functionality (dependent variable). Β-coefficients and 95% confidence interval (95% CI) were performed to present the results. The significance level of 5% was considered statistically significant. Statistical analyzes were performed with IBM SPSS Statistics for Windows, Version 25.0 (Released 2017. IBM Corp., Armonk, New York).

Ethics

The study was accepted by the Ethics Committee of the Scientific Council of the Bioclinic Hospital (number 2870/7-12-2020) and was carried out in accordance with the Declaration of Helsinki (1989) of the World Medical Association. Patients who met the criteria were informed in writing by the researchers about the purposes of the study, the anonymity of the data, that the data will be used only for the study, and that they can withdraw from the study at any time. Patients’ written consent to participate in the study was taken. 

## Results

The basic characteristics of the sample are presented in Table 1. In particular, 57% of the sample were men, while 66% were over 50 years old. Of the participating patients, 55% were married, 54% were high school graduates, and 43% were pensioners.

**Table 1:** Basic demographics of the patients (Ν=100) N: Number

	Ν (%)
Gender	
Male	57(57.0%)
Female	43(43.0%)
Age (years)	
<30	5(5.0%)
30-40	7(7.0%)
41-50	22(22.0%)
51-60	30(30.0%)
61-70	15(15.0%)
>70	21(21.0%)
Marital Status	
Single	55(55.0%)
Married	27(27.0%)
Divorced	8(8.0%)
Widowed	6(6.0%)
Living Together	4(4.0%)
Education Level	
Primary School	18(18.0%)
High School	54(54.0%)
University	26(26.0%)
MSc-PhD	2(2.0%)
Job	
Unemployed	8(8.0%)
Civil Servant	10(10.0%)
Private Employee	15(15.0%)
Freelancer	8(8.0%)
Household	9(9.0%)
Pensioner	43(43.0%)
Other	7(7.0%)

As for data related to the underlying disease and the current state of health are concerned, 99% of the patients were very or enough informed about their health status, 58% described themselves as anxious, and 55% had a lot or enough anxiety about the course of the disease. Also, 34% believed that regular updating helps a lot in reducing stress, 79% had a very good relationship with the nursing staff. while 50% of patients underwent HD for more than four years (median).

The maximum percentage of independence was presented in Drainage Control (98%) followed by Feeding (92%) and Private Toilet (92%) while the lower was presented in the Going up and down the stairs (62%). In total, Severe Dependence (21-60 score) was referred by 6%, Moderate Dependence (61-90) by 17%, and Mild Dependence by 77% of the participants. The median value of functionality was 100 (ΙQR 95-100). Regarding adherence, the median value was 7 (IQR: 7-7) while 77% had scored 7. This indicates high levels of patient adherence. Regarding depression, the median was 38, at least 50% of patients scored < 38, and 25% scored < 34. These values indicate moderate to low levels of patient depression (Table 2).

**Table 2:** Descriptive characteristics of depression, adherence, and functionality in patients undergoing HD (Ν=100). GR-SMAQ-HD: Greek Simplified Medication Adherence Questionnaire; SDS: Self-Rating Depression Scale; N: Number; HD: Hemodialysis; IQR: Interquartile Range

	Median (IQR)
Total GR-SMAQ-HD Score (Range 0-8)	7(7-7)
4	4(4.0%)
5	9(9.0%)
6	7(7.0%)
7	77(77.0%)
Total of Zung SDS score (Range 20-80)	38(34-41.5)
Total Barthell Index Score (Range 0-100)	100(95-100)

The associations of scores on depression, functionality, and adherence are shown in Table 3. A statistically significant negative association emerged between patients' depression score and functionality (r=-0.342, p=0.001) meaning that higher functionality scores indicate lower depression scores. No significant association was obtained between depression and adherence, as well as adherence and functionality.

**Table 3:** Association between depression, functionality, and adherence of patients undergoing HD (N=100) GR-SMAQ-HD: Greek Simplified Medication Adherence Questionnaire; SDS: Self-Rating Depression Scale; N: Number; HD: Hemodialysis

	Zung SDS Score		Barthell Index Score	
	r	p	r	p
Zung SDS Score	-	-	-	-
Barthell Index Score	-0.342	0.001	-	-
GR-SMAQ-HD Score	0.021	0.836	0.078	0.439

Bivariate analyzes showed a statistical relationship at the level of 0.20 (p <0.20) between independent variables (patient characteristics) and depression (dependent variable). For this reason, multivariate linear regression was applied. Female patients had 3.6 points statistically significantly higher depression score than men (β = 3.65, 95% CI: 1.51-5.80, p = 0.001). In addition, patients > 70, patients aged 61-70, and patients aged 51-60 had statistically significantly higher depression scores of 3.5, 4.3, and 2.7, respectively (β = 3.51, 95% CI: 0.09-6.93, p = 0.044, β = 4.26, 95% CI: 1.00-7.52, p = 0.011 and β = 2.72, 95% CI: 0.13-5.31, p = 0.039, respectively) than patients <50 years of age (Table 4).

**Table 4:** Effect of patients’ characteristics and functionality on depression (dependent variable: depression)

	β coefficient (95% CI)	p
Gender		
Male	Reference category	
Female	3.65 (1.51-5.80)	0.001
Age (years)		
≤50	Reference category	
51-60	2.72 (0.13-5.31)	0.039
61-70	4.26 (1.00-7.52)	0.011
>70	3.51 (0.09-6.93)	0.044
Total Barthell Index Score		
Severe / Moderate Dependence	Reference category	
Mild Dependence	-2.15 (-5.12-0.83)	0.155

Bivariate analyzes revealed a statistical relationship at the level of 0.20 (p <0.20) between patient characteristics and depression (independent variables) and functionality (dependent variable). For this reason, multivariate linear regression was applied. Patients over the age of 70 had statistically significantly lower functionality scores of 13.6 points (β = -13.58, 95% CI: -21.68-5.49, p = 0.001). One point increase in patient’s depression indicated a decrease of 0.6 points in patient’s functionality (β = -0.62, 95% CI: -1.06--0.20, p = 0.005) (Table 5).

**Table 5:** Effect of patients’ characteristics and depression on functionality (dependent variable: functionality)

	β coefficient (95% CI)	p-value
Age (years)		
≤50	Reference category	
51-60	1.23(-5.61-8.07)	0.722
61-70	-2.45(-10.52-5.61)	0.547
>70	-13.58(-21.68-5.49)	0.001
Status		
Married / Living Together	Reference category	
Single	-1.22(-8.89-6.45)	0.752
Divorced / Widowed	2.48(-4.68-9.65)	0.492
Education Level		
Primary School	Reference category	
High School	5.58(-1.23-12.38)	0.107
University/ MSc-PhD	5.94(-1.94-13.82)	0.137
Job		
Unemployed/ Household	Reference category	
Employee	2.01(-4.98-9.01)	0.568
Pensioner	2.77(-4.67-10.21)	0.460
Number of children		
0	Reference category	
1		
>1		
Total Depression Score	-0.62(-1.06--0.20)	0.005

## Discussion

This study was conducted in a private hospital in Athens, Greece, aiming to investigate the levels of adherence, functionality, and depression among patients undergoing HD, and the relationship between these variables. The impact of these health issues is significant, as the high depressive symptomatology and the low adherence and functionality may have a negative impact on patients’ outcomes and rise the mortality. As far as we know, this study is the only one that investigates these variables simultaneously in the same sample and, therefore, it is not possible to compare the results with those of other studies. However, other studies have investigated the above variables separately highlighting the high levels of depressive symptoms as well as the significant association of non-adherence and functional insufficiency with depression [6,8].

This study showed moderate to low levels of depression and high levels of adherence and functionality in the total sample of patients undergoing HD. Although these results seem encouraging, in-depth statistical analysis has shown significant differences in depression and functionality levels between men and women and between younger and older patients. Women, in particular, had statistically significantly higher depression scores than men. This finding is similar to those of other studies [24]. Thus, female patients frequently have a negative perception of their mental health. At the same time, aging seems to have a negative effect on the way patients perceive their physical and mental health; patients over the age of 70, patients aged 61-70, and patients aged 51-60 had statistically significantly higher depression scores than patients <50 years of age. It seems that age is one of the strongest predictors of depression among HD patients as it is also referred to in other studies [24]. This finding is probably explained by the cognitive impairment that accompanies old age and, also, by the hormonal dysfunction of kidney disease.

Regarding the effect of age on functionality, the results showed that patients over the age of 70 had statistically significantly lower functionality scores. According to the literature, elderly patients have high rates of functional disability, hospitalization, and mortality from chronic dialysis [5,20]. It seems that the onset of dialysis is directly related to the acceleration of the functional decline.

Studying the relationship between depression and functionality among HD patients, a statistically significant negative association was observed. Specifically, the higher functionality scores indicate lower depression scores. In addition, multivariate linear regression revealed that a one-point increase in patients’ depression indicated a decrease of 0.6 points in patients’ functionality. According to previous studies, it seems that higher levels of functionality are probably associated with lower depression levels [24,25]. A study exploring the association between functional capacity and mental health disorders among HD patients concluded that participants with moderate/severe depression experienced lower levels of functional capacity compared to those with mild/no depression [24]. The same finding was reached by other researchers who, using the ΒΙ, concluded that there was a negative correlation between depression and the ability for daily activities meaning that the higher the level of independence the lower level of depression [25]. This negative correlation is probably due to the fatigue that patients feel due to anemia, malnutrition, inflammation, and metabolic disorders. It is known that fatigue may affect a person's ability to perform daily activities and therefore a decrease in independence leads to an increase in depression levels [24].

In this study, the investigation of the relationship of functionality with adherence didn’t reveal a statistically significant correlation. Although HD adherence and functionality have been extensively studied separately [5,13], there is a lack of simultaneous study of both variables in the same sample of HD patients. Following the search, only two studies emerged: a quantitative and a qualitative. In the quantitative study using self-administered questionnaires, researchers found a positive relationship between adherence to HD regimen and self-care behavior [26]. In particular, the higher the self-care, the higher the adherence while self-care behavior affected positively adherence by 61.3%. On the contrary, in a qualitative study involving Mexican-American women on HD, researchers found minimal differences in functional ability between adherent and non-adherent HD patients [27].

In this study, no statistically significant correlation was found between self-reported adherence to HD regimen and depression. This finding contradicts the findings of previous studies highlighting the significant negative association of depression with adherence [28]. Cukor et al. reported that depression may act as an independent factor of non-adherence in patients undergoing HD [28]. Researchers from Iran using the SMAQ scale to assess medication adherence and the Beck Depression Inventory to assess depression’s levels concluded that non-adherence was higher in people with depressive symptoms [29]. The ambiguous findings may be due to the use of biological indicators (serum phosphate levels). Another explanation that could be given is that our finding may be related to the high rate of functionality on the ΒΙ scale. This explanation is also supported by the finding of another study in which even authors found a negative correlation between depression and adherence to fluid restrictions; when self-care was included in the linear regression model, there was no strong significant correlation between these two variables [30]. In addition, the literature highlights the positive contribution of self-care in the treatment of depression. Therefore, we can conclude that there may not be a causal relationship between depressive symptoms and non-adherence.

Strengths and limitations

The advantage of this study is that it is, to the best of our knowledge, the first that investigates these variables (adherence, functionality, and depression) in the same sample. Regarding the limitations, conducting research during the coronavirus disease 2019 (COVID-19) pandemic and using facemasks may have influenced researchers' communication with patients. Also, the presence of other patients and staff (doctors, dieticians, bankers) in the HD unit may have affected the way patients responded. In addition, the results cannot be generalized since the sample comes from only one HD unit, although Athens is the capital city of Greece and covers all social classes. Moreover, there was a time limit as the questionnaires had to be completed during the HD session. For this reason, those patients who did not catch up were given the opportunity to take the questionnaires home and return them to the next session.

## Conclusions

The results of the current study indicate moderate to low levels of patients’ depression, high levels of adherence, and functionality. As far as the relationship between depression and functionality among HD patients is concerned, it was found that the higher the levels of functionality, the lower the levels of depression. Regarding the relationship between depression and adherence, as well as adherence and functionality, no significant association was observed. As for demographic characteristics, age (>70years old) and gender (women) seem to be significant predictors of depression and functionality. Therefore, recognizing and treating depression by healthcare professionals may improve the levels of functionality and reduce patients’ dependence leading to better health outcomes.

## Appendices

**Table 6:** The Greek-Simplified Medication Adherence Questionnaire-Hemodialysis (GR-SMAQ-HD)

(Τhe numbers in parenthesis is the score)
Medication Adherence
When you feel bad, have you ever stopped taking your medications? Yes (0) No (1)
Have you ever forgotten to take your medications? Yes (0) No (1)
Have you ever forgotten to take your medications on the days between the two dialysis sessions? Yes (0) No (1)
In the last week, how many times have you not taken your medications? Never (1) 1-2 times (1) 3-5 times (0) 6-10 times (0) Over 10 times (0)
Attendance at Hemodialysis Session
Last month, how many times was the session shortened on your own initiative? Ι have not shortened the session (1) Once (1) Two times (0) Three times (0) Four-five times (0)
Last month, on average, how many minutes was the session cut off on your own initiative? I have not shortened the session (1) 10 minutes or less (1) 11-20 minutes (0) 21-30 minutes (0) Over 30 minutes (0)
Fluid/Diet
During the past week, how many times did you follow fluid restrictions? Every time (1) Most of the times (1) About half the times (0) Rarely (0) Never (0)
During the past week, how many times did you follow dietary recommendations? Every time (1) Most of the times (1) About half the times (0) Rarely (0) Never (0)

**Table 7:** The Barthel Index for assessing the functionality

ACTIVITY	SCORE
Feeding	0 = unable
	5 = needs help cutting, spreading butter, etc., or requires modified diet
	10 = independent
Bathing	0 = dependent
	5 = independent (or in shower)
Grooming	0 = needs to help with personal care
	5 = independent face/hair/teeth/shaving (implements provided
Dressing	0 = dependent
	5 = needs help but can do about half unaided
	10 = independent (including buttons, zips, laces, etc.)
Bowels	0 = incontinent (or needs to be given enemas)
	5 = occasional accident
	10 = continent
Bladder	0 = incontinent, or catheterized and unable to manage alone
	5 = occasional accident
	10 = continent
Toilet use	0 = dependent
	5 = needs some help, but can do something alone
	10 = independent (on and off, dressing, wiping)
Transfers (bed to chair and back)	0 = unable, no sitting balance
	5 = major help (one or two people, physical), can sit
	10 = minor help (verbal or physical)
	15 = independent
Mobility (on level surfaces)	0 = immobile or < 50 yards
	5 = wheelchair independent, including corners, > 50 yards
	10 = walks with help of one person (verbal or physical) > 50 yards
	15 = independent (but may use any aid; for example, stick) > 50 yards
Stairs	0= unable
	5= needs help (verbal, physical, carrying aid)
	10= independent
Total	0-100

**Table 8:** The Zung Self-Rating Depression Scale (SDS)

	A little of the time	Some of the time	Good part of the time	Most of the time
I feel down-hearted and blue				
Morning is when I feel the best				
I have crying spells or feel like it				
I have trouble sleeping at night				
I eat as much as I used to				
I still enjoy sex				
I notice that I am losing weight				
I have trouble with constipation				
My heart beats faster than usual				
I get tired for no reason				
My mind is as clear as it used to be				
I find it easy to do the things I used to				
I am restless and can’t keep still				
I feel hopeful about the future				
I am more irritable than usual				
I find it easy to make decisions				
I feel that I am useful and needed				
My life is pretty full.				
I feel that others would be better off if I were dead.				
I still enjoy the things I used to do				

## References

[1] (2022). USRDS 2020 Annual Data. End stage renal disease: Incidence, prevalence, patient characteristics, and treatment modalities. https://adr.usrds.org/2020/end-stage-renal-disease/1-incidence-prevalence-patient-characteristics-and-treatment-modalities.

[2] (2022). National Transplant Agency of Greece: Kidney transplant. The kidneys and their function [Website in Greek]. https://www.eom.gr/metamoscheysi-nefroy/.

[3] Pretto CR, Winkelmann ER, Hildebrandt LM, Barbosa DA, Colet CF, Stumm EM (2020). Quality of life of chronic kidney patients on hemodialysis and related factors. Rev Lat Am Enfermagem.

[4] Elhadad AA, Ragab AZ, Atia SA (2020). Psychiatric comorbidity and quality of life in patients undergoing hemodialysis. Middle East Curr Psychiatry.

[5] Tavares AR Jr, Vieira CP, Souza PA (2010). Functional status of elderly adults receiving dialysis. N Engl J Med.

[6] Khan A, Khan AH, Adnan AS, Sulaiman SA, Mushtaq S (2019). Prevalence and predictors of depression among hemodialysis patients: a prospective follow-up study. BMC Public Health.

[7] Al Garni RS, Cooke M (2021). The concept of HRQoL for patients on hemodialysis in Saudi Arabia: an exploratory study. Health Qual Life Outcomes.

[8] Al Awwa IA, Jallad SG (2018). Prevalence of depression in Jordanian hemodialysis patients. Iran J Psychiatry Behav Sci.

[9] Sabaté E (2003). Defining adherence. Adherence to Long-Term Therapies: Evidence for Action.

[10] Estrella MM, Jaar BG, Cavanaugh KL (2013). Perceptions and use of the national kidney foundation KDOQI guidelines: a survey of U.S. renal healthcare providers. BMC Nephrol.

[11] Ghimire S, Castelino RL, Lioufas NM, Peterson GM, Zaidi ST (2015). Nonadherence to medication therapy in haemodialysis patients: a systematic review. PLoS One.

[12] Parker K, Bull-Engelstad I, Aasebø W, von der Lippe N, Reier-Nilsen M, Os I, Stavem K (2019). Medication regimen complexity and medication adherence in elderly patients with chronic kidney disease. Hemodial Int.

[13] Alikari V, Tsironi M, Matziou V, Babatsikou F, Psillakis K, Fradelos E, Zyga S (2018). Adherence to therapeutic regimen in adults patients undergoing hemodialysis: the role of demographic and clinical characteristics. Int Arch Nurs Health Care.

[14] Zung WW (1965). A self-rating depression scale. Arch Gen Psychiatry.

[15] Lam RW, Michalaak EE, Swinson RP (2006). Assessment scales in depression, mania and anxiety. http://files3.moshaverfa.com/en/pdf/Assessment/2005_-_Assessment_scales_in_depression,_mania_and_anxiety_-_Lam,_Michalak,_Swinson.pdf.

[16] Dunstan DA, Scott N, Todd AK (2017). Screening for anxiety and depression: reassessing the utility of the Zung scales. BMC Psychiatry.

[17] Fountoulakis KN, lacovides A, Samolis S, Kleanthous S, Kaprinis SG, St Kaprinis G, Bech P (2001). Reliability, validity and psychometric properties of the Greek translation of the Zung Depression Rating Scale. BMC Psychiatry.

[18] Mahoney FI, Barthel DW (1965). Functional evaluation: the Barthel index. Md State Med J.

[19] Duffy L, Gajree S, Langhorne P, Stott DJ, Quinn TJ (2013). Reliability (inter-rater agreement) of the Barthel Index for assessment of stroke survivors: systematic review and meta-analysis. Stroke.

[20] Cook WL, Jassal SV (2008). Functional dependencies among the elderly on hemodialysis. Kidney Int.

[21] Theofanidis D (2017). Validation of international stroke scales for use by nurses in Greek settings. Top Stroke Rehabil.

[22] Alikari V, Matziou V, Tsironi M (2017). A modified version of the Greek Simplified Medication Adherence Questionnaire for hemodialysis patients. Health Psychol Res.

[23] Alikari V, Tsironi M, Matziou V (2019). The impact of education on knowledge, adherence and quality of life among patients on haemodialysis. Qual Life Res.

[24] Liu YM, Chang HJ, Wang RH, Yang LK, Lu KC, Hou YC (2018). Role of resilience and social support in alleviating depression in patients receiving maintenance hemodialysis. Ther Clin Risk Manag.

[25] Santacruz J, Santacruz S, Coloma G (2021). Functional impairment associated with increased severity of mental health disorders in Latin American chronic hemodialysis patients. Arch Renal Dis Manag.

[26] Kim H, Cho MK (2021). Factors influencing self-care behavior and treatment adherence in hemodialysis patients. Int J Environ Res Public Health.

[27] Tijerina MS (2009). Mexican American women's adherence to hemodialysis treatment: a social constructivist perspective. Soc Work.

[28] Cukor D, Rosenthal DS, Jindal RM, Brown CD, Kimmel PL (2009). Depression is an important contributor to low medication adherence in hemodialyzed patients and transplant recipients. Kidney Int.

[29] Ossareh S, Tabrizian S, Zebarjadi M, Joodat RS (2014). Prevalence of depression in maintenance hemodialysis patients and its correlation with adherence to medications. Iran J Kidney Dis.

[30] Washington TR, Hain DJ, Zimmerman S, Carlton-LaNey I (2018). Identification of potential mediators between depression and fluid adherence in older adults undergoing hemodialysis treatment. Nephrol Nurs J.

